# Unified Formulation for a Triaxial Elastoplastic Constitutive Law for Concrete

**DOI:** 10.3390/ma6094226

**Published:** 2013-09-23

**Authors:** Rabah Hammoud, Rachid Boukhili, Ammar Yahia

**Affiliations:** 1 École Polytechnique de Montréal, Montréal, Québec H3C 3A7, Canada; E-Mail: rachid.boukhili@polymtl.ca; 2 Université de Sherbrooke, Sherbrooke, Québec J1K 2R1, Canada; E-Mail: ammar.yahia@usherbrooke.ca

**Keywords:** constitutive law, elasto-plastic material, failure criterion, finite element method, triaxial strength

## Abstract

A constitutive model to describe the triaxial load-response spectrum of plain concrete in both tension and shear was developed. The inelastic phenomena are described using the plastic flow with direction determined by the gradient of the plastic potential. A new plastic potential is introduced and experimentally fitted to ensure better estimate of the load direction. This approach allows to control the inelastic dilatancy in terms of the inelastic deformation of the material. By overlaying the plastic potential on modified Etse and Willam’s yield surface (both defined on the Haigh–Westergaard coordinates), the results showed that the two curves do not undergo similar stress states for a given strength level. It is, therefore, necessary that each surface goes through the current stress state to ensure adequate evaluation of normal vectors. A closed-form solution to accurately predict the triaxial stress state in concrete has been proposed. The predictive capabilities of the proposed model are evaluated by comparing predicted and measured stresses. The proposed model is shown to be accurate in predicting stress state of concrete.

## 1. Introduction

Mechanical behavior of materials is generally analyzed based on conditions associated with particular states, such as the yield stress, the limits in compression, and the post-peak behavior. In order to describe the evolution of these states, scalar functions describing failure criteria were developed [1]. These functions are expressed in the space of principal stresses to reflect the physical evolution of the materials [1]. Various studies conducted on the behavior of concrete showed that loading mode generates transitions in its behavior [2]. For instance, a material can change from elastic to inelastic or from pre-rupture to post-rupture phase. In order to adequately represent the response of a material under different loads, the constitutive laws should therefore take into account the specific conditions related to these transitions. Criteria of plasticity (or of flow) which are convex in the space of the principal stresses are then introduced.

Various studies have been published on plasticity models for concrete [3,4,5,6,7,8,9,10]. In general, cohesive and frictional materials, such as concrete, exhibit complex responses, including pressure sensitivity, inelastic dilatancy, strain softening, and path dependency [11]. These are the key factors responsible of the nonlinear behavior of concrete. The main difficulty in developing models that can accurately describe the behavior of concrete is the strong dependence of the strength, the stiffness, and the ductility with the load path. The development of plasticity criteria followed two major approaches that are usually applied with metals and geomaterials [3]. The majority of the models employed for porous metal compounds and powders are based upon the von Mises criterion [12]. For example, Aubertin *et al.* [12] used criteria independent of the stress’s first invariant $I_{1}$ (or mean stress $\sigma_{m}=I_{1}/3$) as it is in the case of the criterion of Tresca and von Mises. The frictional component associated with the effect of spherical part (or hydrostatic) of $\sigma_{ij}$ is neglected. On the other hand, the Coulomb criterion was the basis for the majority of the criteria employed for the geomaterials (rocks, concretes, *etc*).

Drucker and Prager proposed a circular version in the plane of the octahedral stresses (*i.e*., near to von Mises criterion), while maintaining the linear relation between $I_{1}$ and $J_{2}^{1/2}$ (without using *θ* or $J_{3}$) [13]. Comparative syntheses and criticisms on these various criteria were presented by Chen [14]. Lade [15,16,17] carried out latter three dimensional compressive tests on non-cohesive soils to determine their mechanical resistance characteristics. Test results revealed that the failure surfaces resemble to the deviatoric sections of Mohr–Coulomb model in the deviatoric plan except that they are always smooth and regular [16]. Thereafter, the authors developed a function of the first invariant $I_{1}$ and the third invariant $I_{3}$ of the stress tensor. Schreyer [18] proposed a failure surface in terms of the three invariants of stress, in which the form of surface in the deviatoric plane is a function of the mean pressure. At low mean pressure, the shape of section is a triangle, but it changed to circular shape with higher pressure levels. This is similar to that of Drucker–Prager at high pressure levels. The model seems to be applicable to steels, geotechnical materials, and concrete [18].

A unified elastoplastic model for concrete with strain hardening and softening using the Willam and Warnke failure criterion with a non-associated flow rule was developed [19,20]. The plastic potential takes the form of a Druker–Prager to enable direct assessment of the normal potential.

The models developed by Pramono and Willam [21] and by Etse and Willam [22] reproduced well the three dimensional deformations and stresses of concrete under various loads. The failure surface is a function of three invariants of stress tensor and has curved meridians and trilobate deviatoric sections. The elastoplastic model has a system of hardening and softening parameters. Equivalent plastic strain serves as hardening variable and itself is a function of pressure. Although the model can accurately assess the magnitude of permanent deformation under various loads, it did not identify plastic potential [21,22]. To estimate the direction of strain, the derivative of the function of the loading surface is therefore changed.

Crouch and Tahar [23] took over the model of Etse and Willam [22] and introduced a new plastic potential to better reproduce the direction of plastic deformation. They changed the functions of softening in order to take into account the contribution of failure mode on the rate of released energy. Other researchers modified the Menetrey and Willam [6] model by changing the hardening function, which became a function of the volumetric plastic strain instead of the equivalent plastic strain [9,10]. The modified model is shown to better predict the stresses and strains for uniaxial, biaxial, and triaxial compression loading modes. Meyer *et al.* [24] presented an elastoplastic model covering the nonlinear triaxial behavior of the concrete under both compression and tension loading. Since the behavior in tension is different from that in compression, two different functions of hardening are defined for tension and compression loading modes, respectively. The surface of loading evolves (*i.e*., moves) according to a factor *k*, which is different according to the loading mode (tension or compression). To model the nonlinear behavior of the volumetric contraction-dilation, a non-associated law of flow is applied. In addition to avoid tensile stress, those models should be properly combined with a tensile fracture model to allow their implementation in general finite element applications.

Although prominent studies and attempts are made to improve different constitutive models, it should be noted that the suggested models are only applicable in the concrete compression regime or using nonspecific hardening-softening functions. The main objective of this study is to formulate an extent triaxial constitutive model that can successfully simulate the large spectrum of loading. The proposed model should be efficiently implemented in a finite element code.

## 2. Triaxial Constitutive Formulation

Reliable finite element modeling of concrete requires the use of accurate constitutive models. Although reliable models exist, their inability to take into account concrete plasticity, which is necessary in modeling actively-confined concrete behavior, limits their usefulness to represent general states of stress. The concrete plasticity needs to include the following three features [14]: (*a*) a yield criterion, including the third deviatoric stress invariant; (*b*) a hardening/softening rule, which is dependent on the confining pressure; and (*c*) a flow rule, which is dependent not only on the confining pressure but also on the confinement increment.

### 2.1. Yield Surface Criterion

Concrete failure must encompass pressure sensitivity and tensile-strength limiting [14]. This is the consequence of the combined effect of cohesive strength of cement paste and frictional adhesion of aggregate interlock. Beyond the elastic limit, concrete can break in tension, shear, or confined compression. The model proposed by Etse and Willam [22] to describe the triaxial behavior under a wide range of loading histories is modified to take into account the elastic-plastic behavior of concrete. The resulting strength criterion provides a fair representation of the tensile/cohesive strength of cement materials and a reasonable description of shear-strength. The proposed failure criterion is expressed using Haigh–Westergaard coordinates, which span a cylindrical coordinate system in the stress space. This criterion is given by:

(1) $$F\left(\sigma_{m},\rho ,r\left(\theta \right)\right)=\frac{3}{2}{\left[\frac{\rho \;r\left(\theta \right)}{f_{c}}\right]}^{2}+\frac{m_{f}}{f_{c}}\left[\sigma_{m}+\frac{\rho \;r\left(\theta \right)}{\sqrt{6}}\right]-1=0$$

The three unified coordinates $\sigma_{m}$, *ρ*, and *θ* are defined as:

- $\sigma_{m}$ is the mean normal stress or hydrostatic pressure expressed by:
  (2) $$\sigma_{m}=\frac{I_{1}}{3}=\frac{tr(\sigma )}{3}$$
- *ρ* is the deviatoric stress defined by:
  (3) $$\rho =\sqrt{2J_{2}}$$
  $J_{2}$ is the second invariant of the deviatoric stress tensor *s*:
  (4) $$s_{ij}=\sigma_{ij}-\sigma_{m}\delta_{ij}$$
  (5) $$J_{2}=\frac{1}{2}s_{ij}s_{ij}$$
- *θ* is the polar angle that determines the direction of the octahedral shear stress and locates the stress state relative to the meridians of tension and compression around the hydrostatic axis. The angle *θ* is defined as follows:
  (6) $$\cos3\theta =\frac{\sqrt{27}}{2}\frac{J_{3}}{J_{2}^{\frac{3}{2}}}$$
  $J_{3}$ is the third invariant of the deviatoric stress tensor *s* defined by:
  (7) $$J_{3}=s_{ij}s_{jk}s_{ki}=det\;s$$

The parameter $m_{f}$ is the ratio between the compressive strength ($f_{c}$) and the triaxial tensile shear value ($f_{tt}$) as follows:

(8) $$m_{f}=\frac{f_{c}}{f_{tt}}$$

Triaxial tensile strength $f_{tt}$ is assumed equal to the uniaxial tensile strength value [22]. The triaxial criterion can be approximated by an elliptic description of the Willam and Warnke model in the deviatoric region to generate a continuous surface [25]. The polar coordinate is expressed as [25]:

(9) $$r\left(\theta ,e_{r}\right)=\frac{4\left(1-e_{r}^{2}\right)\cos^{2}\theta +{\left(2e_{r}-1\right)}^{2}}{2\left(1-e_{r}^{2}\right)\cos\theta +\left(2e_{r}-1\right)\sqrt{4\left(1-e_{r}^{2}\right)\cos^{2}+5e_{r}^{2}-4e_{r}}}$$

The eccentricity ratio $e_{r}$ is defined as the ratio of the tensile meridian to the compressive meridian [25]:

(10) $$e_{r}=1-\frac{1}{2}\frac{\left(\sigma_{m_{0}}-c_{e}\right)}{\sigma_{m}-c_{e}}$$

where $c_{e}$ and $\sigma_{m_{0}}$ are constants. The term $\sigma_{m_{0}}$ is the triaxial tensile strength of the material. To preserve convexity, the eccentricity must be between 0.5 and 1 (0.5≤e≤1) [22].

**Figure 1:**
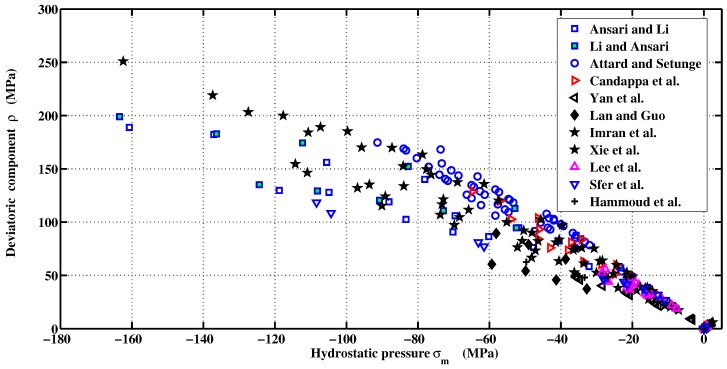
Experimental data on Haigh–Westergaard coordinates.

The variation of deviatoric components with hydrostatic pressure taken from literature is presented in Figure 1 (Li and Ansari [26], Ansari and Li [27], Candappa *et al.* [28], Imran and Pantazopoulou [29], Sfer *et al.* [30], Xie *et al.* [31], Yan *et al.* [32], Attard and Setunge [33], Lan and Guo [34], Lee *et al.* [35], Hammoud *et al.* [36]). These various studies were carried out by taking into account the degree of concrete saturation, type of cement and aggregates, loading path, dimension of specimens, confinement levels, *etc*. For this reason, the failure criterion was originally formulated as an expression of second order parabolic Mohr envelope [22]. The proposed yield surface can be represented by a more flexible approximation given by Equation (11):

(11) $$F\left(\sigma_{m},\rho ,r\left(\theta \right)\right)=a_{f}{\left[\frac{\rho \;r\left(\theta \right)}{f_{c}}\right]}^{\alpha_{f}}+\frac{m_{f}}{f_{c}}\left[\sigma_{m}+\frac{\rho \;r\left(\theta \right)}{b_{f}}\right]-1=0$$

where $r\left(\theta \right)$ is the polar coordinate; $f_{c}$ is the absolute value of uniaxial compression strength; and $a_{f}$ , $m_{f}$, $b_{f}$ are constants. The variable $\alpha_{f}$ is used to define $\rho (\sigma_{m})$ as a nonlinear function.

**Figure 2:**
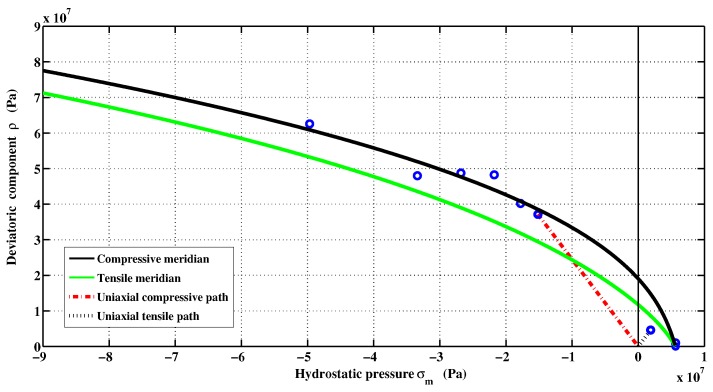
Meridional sections of triaxial failure.

**Figure 3:**
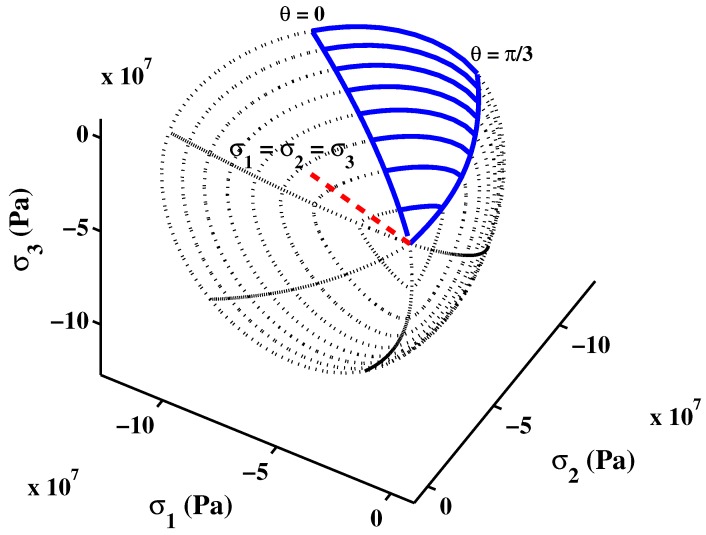
Deviatoric sections of triaxial failure.

The failure surface is plotted in Figure 2 for the meridional sections θ=0 (tensile meridian), and θ=π/3 (compressive meridian). On the other hand, the deviatoric sections at different levels of mean normal stress are shown in Figure 3. As can be observed, the deviatoric sections approach the triangular shape of the Rankine envelope in tension mode. The shape becomes circular approaching Drucker–Prager criterion for higher confinement. The depicted failure is smooth and a $C^{1}$-continuous curvilinear trace.

### 2.2. Isotropic Loading Surfaces in Pre- and Post-Peak

#### 2.2.1. Isotropic Hardening

In the present formulation, two assumptions were made: (1) The concrete is isotropic and remains isotropic during the entire deformation process; and (2) the elastic-plastic coupling is neglected. During the hardening regime, the loading surfaces are generated by individual specific values of normalized strength parameter *k*, where 0≤k≤1. At the same time, the cohesion parameter *c* related to the softening regime remains constant during loading. At the beginning of loading, the elastic regime is limited by a surface loading with initial value of $k=k_{0}$. The function of the failure envelope in Equation (11) is then modified to take the following form:

(12) $$F\left(\sigma_{m},\rho ,r,k,c\right)\;=\;{\left\{\left(1-k\right){\left[\frac{\sigma_{m}}{f_{c}}\;+\;\frac{\rho \;r\left(\theta \right)}{b_{f}f_{c}}\right]}^{2}\;+\;a^{\frac{1}{\alpha_{f}}}\frac{\rho \;r\left(\theta \right)}{f_{c}}\right\}}^{\alpha_{f}}\;+\;\frac{k^{\beta_{f}}m_{f}}{f_{c}}\left[\sigma_{m}\;+\;\frac{\rho r\left(\theta \right)}{b_{f}}\right]-k^{\beta_{f}}c=0$$

**Figure 4:**
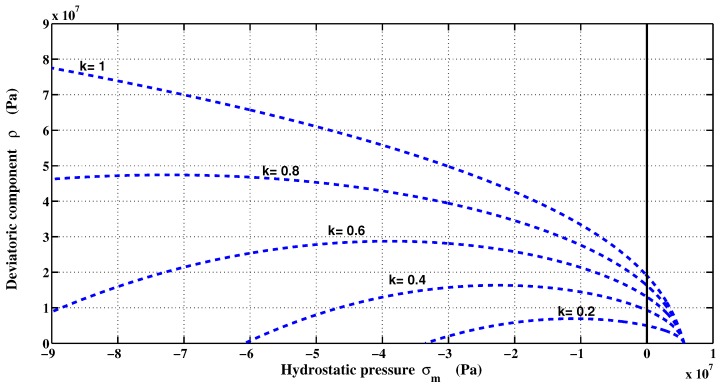
Triaxial failure envelope in hardening mode.

The Equation (12) defines the surface loading, which is highly important in the plastic model. This was initially formulated by Etse and Willam [22], except that the parameter $\beta_{f}$ is introduced instead of $k^{2}$ in the original model. This parameter was first introduced for carbonaceous materials [37]. The originality of the proposed triaxial failure function consists in reproducing the hardening regime of the material in both tension and compression by incorporating $\beta_{f}$ to reproduce experimental stress-strain (*σ*-*ϵ*) curves (e.g., [7,23]). The shape of the loading surface is shown in Figure 4. The loading surface is then closed to define a certain elastic region. It is assumed that the material begins its hardening process at 20% of its ultimate strength. Through the uniaxial compression path, the hardening is linear because the distance between two successive surfaces is kept the same. In uniaxial tension, the hardening is not linear because the surface at k=0.8 and k=1 are superimposed.

#### 2.2.2. Isotropic Softening

**Figure 5:**
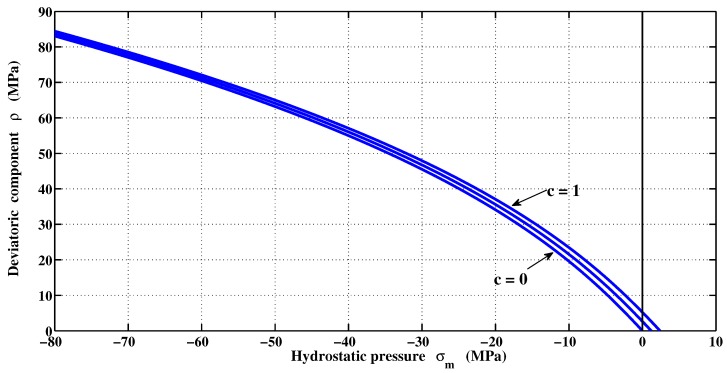
Triaxial failure envelope in softening mode.

In order to obtain a continuous model taking into account the reduction of strength in a rational manner, the plastic-softening model adopt the concept of fracture energy to assess the strength degradation in both tension and compression loading modes. The fracture energy is expressed in terms of crack opening (mode I) and is extended to the splitting in shear and compression (mode II) as well as distributed microcracking in shear (mode II and mode III). The failure mode depends mainly on the level of confinement where the softening is most pronounced in direct tension. The loading function (Equation (12)) is modified to describe the degradation of the tensile strength and shear in the form of isotropic decohesion. When the cohesion parameter *c* decreases to zero, the values of tensile strength also tend to zero. At this stage (c=0), the residual resistance may be due to friction between aggregates and paste matrix. The surface failure during softening varies depending on the decohesion and is expressed by Equation (13) (see also Figure 5):

(13) $$F_{s}\left(\sigma_{m},\rho ,r\left(\theta \right),c\right)=a_{f}{\left[\frac{\rho \;r(\theta )}{f_{c}}\right]}^{\alpha_{f}}+\frac{m_{f}}{f_{c}}\left[\sigma_{m}+\frac{\rho \;r\left(\theta \right)}{b_{f}}\right]-c=0$$

## 3. Plastic Potential Function

Based on the strain decomposition into elastic $\epsilon_{e}$ and plastic $\epsilon_{p}$ components [38], the total strain is expressed as follows:

(14) $$\dot{\epsilon }={\dot{\epsilon }}_{e}+{\dot{\epsilon }}_{p}$$

The elastic response of the material is defined by Hooke’s law using the elasticity tensor *H*. The plastic response is governed by flow rule:

(15) $${\dot{\epsilon }}_{p}=\dot{\lambda }\frac{\partial Q}{\partial \sigma }=\dot{\lambda }m$$

where *Q* denotes the plastic potential and *λ* is the plastic multiplier. Plastic flow rule specifies the rate of change in the plastic deformation at a controlled stress event. To reduce excessive dilatation in the low confinement region, a non-associated flow rule is introduced. A new plastic potential is then defined by re-using the loading function and replacing the set of parameters $a_{f}$, $b_{f}$, and $\alpha_{f}$ by new ones named $a_{q}$, $b_{q}$ and $\alpha_{q}$. The plastic potential function is given by Equation (16):

(16) $$Q\left(\sigma_{m},\rho ,r\left(\theta \right)\right)=a_{q}{\left[\frac{\rho \;r(\theta )}{f_{c}}\right]}^{\alpha_{q}}+\frac{m_{f}}{f_{c}}\left[\sigma_{m}+\frac{\rho \;r(\theta )}{b_{q}}\right]-1=0$$

To identify the parameters of the plastic potential, it is necessary to know the normal vectors to the potential at rupture for a few cases of loading. The relationship between volumetric and deviatoric components of the normal potential must be the same to that obtained in compression tests. In this case, it is possible to use circular deviatoric sections (with $e_{r}=1$, and r(θ)=1 in Equation (9)) instead of the elliptical sections of the failure envelope. Also, the computing time needed to integrate the constitutive law is shorter. Moreover, the observed difference in terms of stress and strain is negligible. The equation of the plastic potential in the hardening/softening regimes takes the same expression as that of the failure surface:

(17) $$Q\left(\sigma_{m},\rho ,r,k,c\right)\;=\;{\left\{\left(1-k\right){\left[\frac{\sigma_{m}}{f_{c}}\;+\;\frac{\rho r(\theta )}{b_{q}f_{c}}\right]}^{2}\;+\;a^{\frac{1}{\alpha_{q}}}\frac{\rho r(\theta )}{f_{c}}\right\}}^{\alpha_{q}}\;+\;\frac{k^{\beta_{q}}m_{f}}{f_{c}}\left[\sigma_{m}+\frac{\rho r(\theta )}{b_{q}}\right]-k^{\beta_{q}}c=0$$

## 4. Hardening and Softening Parameter Functions

Hardening and softening of concrete can be simulated by varying the shape and location of the loading surface during plastic flow. The strength parameter *k* determines the size of the yield or loading surfaces in the hardening regime before the rupture. This parameter is expressed by quadratic function of the accumulated plastic strain $\epsilon_{p}$ and the ductility $d_{h}$. The function used for *k* is given by Equation (18) [22]:

(18) $$k\left(\epsilon_{p},d_{h}\right)=k_{0}+\left(1-k_{0}\right)\sqrt{\frac{\epsilon_{p}}{d_{h}}\left(2-\frac{\epsilon_{p}}{d_{h}}\right)}$$

This function is formulated to reach k=1 when $\epsilon_{p}/d_{h}=1$. The rate of equivalent plastic strain $\epsilon_{p}$ is determined by the norm of the plastic strain tensor:

(19) $${\dot{\epsilon }}_{p}=\dot{\lambda }∥m∥$$

**Figure 6:**
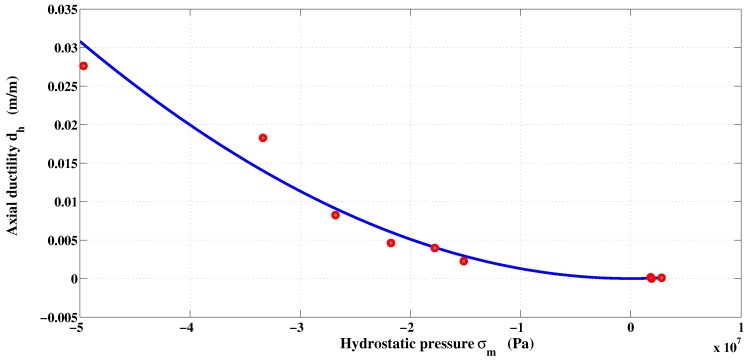
Ductility curve *versus* mean pressure.

The measurement of ductility $d_{h}$ is used to take into account the influence of confinement on the material’s ability to deform permanently. It defines the maximum equivalent plastic strain when the failure envelope is reached. The failure is obtained when the condition $\frac{\epsilon_{p}}{d_{h}}=1$ is satisfied. Ductility curve is constructed from the equivalent plastic strain at failure obtained during tensile tests, uniaxial compression, and confined compression. Since only the equivalent plastic strain of the uniaxial compression and compression with confinement tests are known, further calculated values must be obtained. The relationship between the ductility and the pressure is shown in Figure 6. Two analytic functions are necessary to reproduce the curve of ductility and to separate the tensile and compression strains [37]. The functions used by Etse and Willam [22] and by Kang and Willam [8] cannot properly evaluate the plastic strain in tension, because they are generally polynomials of degree two or three. The function proposed for ductility is:

(20) $$d_{h}=\left\{\begin{array}{cc} \frac{1}{a_{d}+b_{d}\sigma_{m}^{c_{d}}} & \text{for}\;\sigma_{m}\geq 0\\ \exp\left(e_{d}+\frac{f_{d}}{|\sigma_{m}|}+g_{d}\ln\left(|\sigma_{m}|\right)\right)+h_{d} & \text{for}\;\sigma_{m}<0 \end{array}\right.$$

The superposition of these two functions provides a curved shape of the relation $d_{h}\left(\sigma_{m}\right)$ in the region of the uniaxial tension similar to that in Figure 6. Parameters optimization is carried out by non-linear regression fitting. The optimized parameters are: $a_{d}=-413.2128$, $b_{d}=935.1668$, and $c_{d}=0.1765$. On the other hand, the values of $e_{d}$, $f_{d}$, $g_{d}$ and $h_{d}$ are -26.8414, -21.51e-06, 1.3410, and 8.71e-5, respectively.

The softening phenomenon is defined as a gradual decrease of the mechanical resistance during a continuously increasing deformation. The material undergoes a gradual internal debonding. An exponential softening function is adopted here as follows [7]:

(21) $$c=\frac{1}{\exp\left(\delta_{s}w^{2}\right)}$$

where *w* is the displacement of the crack opening for a direct tensile test; and $\delta_{s}$ is a constant controlling the rapidity of the Gaussian decay. Considering the invariance of softening compared with samples of different heights, it is possible to combine the resistance degradation with the homogenization of crack opening displacement. This displacement is replaced by a plastic fracture strain $\epsilon_{f}$ in an elastic-plastic equivalent environment to obtain the following equation [23]:

(22) $$\epsilon_{f}=\frac{w}{l_{c}}$$

where $\epsilon_{f}$ refers to the equivalent strain at fracture in tension; and $l_{c}$ is the characteristic length of the material. The characteristic length $l_{c}$ is a measure of the distance between two parallel cracks inside the material. It is related to the heterogeneity size within the material or the aggregates size. According to Crouch and Tahar [23] the characteristic length $l_{c}$ can be determined by Equation (23):

(23) $$l_{c}=2.7d_{a}$$

where $d_{a}$ is the average diameter of the largest aggregate. The incremental fracture strain is defined in terms of the positive components in the Euclidean norm of the plastic strain increment [23]:

(24) $$\Delta \epsilon_{f}=\sqrt{<\Delta \epsilon_{p1}{>}^{2}+<\Delta \epsilon_{p2}{>}^{2}+<\Delta \epsilon_{p3}{>}^{2}}=\Delta \lambda ∥<m>∥$$

where <> are the Macaulay brackets that extract the positive tensile component from the principal plastic increment. The $\epsilon_{f}$ increments are nonzero only if microcracks exist.

The fracture model for Mode I loading tensile cracking described above is extended to encompass Mode II/III type shear fracture. Distributed microcracking occurs under increasing confinement as the Modes II or III fracturing appears. The general crack model can be interpreted as a multiple tensile crack approach. Mixed mode fracturing consider the number of cracks (*N*) that are formed in a specimen under a given state of stress. The resulting amount of fracture energy $G_{f}$ that is dissipated in a specimen is therefore $NG_{f}$. The Lode angle *θ* is included in the model to distinguish between the two failure types. Using the expression from Menetrey and Willam [6], the number of cracks is determined by Equation (25):

(25) $$N=\left\{\begin{array}{cc} 1 & \text{for}\;\frac{\sigma_{m}}{\rho }\geq \frac{1}{\sqrt{6}}\\ \sqrt{2}\left(\frac{-\sqrt{3}\sigma_{m}}{\rho }+\frac{1}{\sqrt{2}}\right)\left(1-\cos\left(\theta +\frac{\pi }{6}\right)\right)\left(N_{uc}-1\right)+1 & \text{for}\;\frac{\sigma_{m}}{\rho }<\frac{1}{\sqrt{6}} \end{array}\right.$$

## 5. Algorithmic Formulation

### 5.1. Evaluation of Convenient Stress for Plastic Potential

By overlaying the plastic potential on the modified Etse and Willam yield surface, both defined on the Haigh–Westergaard coordinates, it can be observed that for a given strength parameter *k*, the two curves do not undergo the same stress states. In order to ensure adequate evaluation of normal vectors, it is necessary that each surface goes through the current stress state. Keeping the loading surface unchanged, the calculation related to the plastic potential needs to be modified. D’amours [37] proposed an original procedure by identifying a new value of deviatoric component *ρ* prior to the evaluation of the gradient of plastic potential, thus allowing a vertical move of the stress state to the plastic potential for Q=0. This method is valid for both hardening and softening modes. However, it is essential to use circular deviatoric sections and analytical derivatives to isolate *ρ*, then modify the calculation of numerical derivatives. To minimize the plastic potential, the new value of *ρ* is obtained using the following iterative relationship:

(26) $$\rho_{i+1}=\rho_{i}-\frac{Q\left(\sigma_{m},\rho_{i},r,k\right)}{\frac{\partial Q\left(\sigma_{m},\rho_{i},r,k\right)}{\partial \rho }}$$

Special attention is given to the calculations of the derivatives. The terms $\frac{\partial Q}{\partial \sigma_{m}}$ and $\frac{\partial Q}{\partial \rho }$ are evaluated with $\rho_{Q}$, while $\frac{\partial \rho }{\partial \sigma }$ and $\frac{\partial \sigma_{m}}{\partial \sigma }$ are evaluated with the real vector of stress. In general, *ρ* is the deviatoric stress component as defined in Equation (3) in this paper. For avoiding ambiguity, we have used $\rho_{F}$ and $\rho_{Q}$ to indicate deviatoric stresses for the yield surface and plastic potential, respectively.

### 5.2. Resolution Scheme

**Figure 7:**
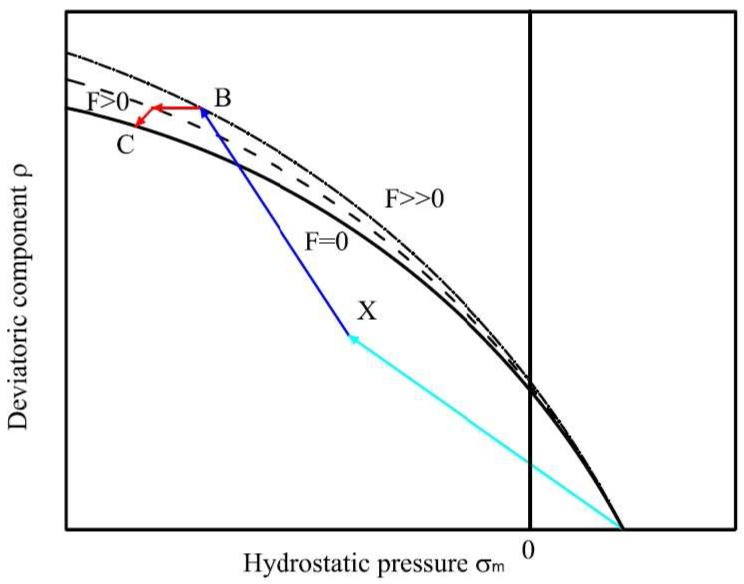
Predictor Elastic test point.

A backward-Euler (Euler implicit) algorithm as defined by Crisfield [39] is applied for constitutive integration. The algorithm for each integration point for a given stress can be summarized by means of the following steps:

- Calculating the first elastic prediction:
  1. From the stresses at point *B* (Figure 7), calculate the value of *F* and the gradient $n=\frac{\partial F\left(\sigma_{m},\rho ,r,k\right)}{\partial \sigma }$.
  2. In the presence of non-associated flow, identify a particular value of *ρ* for Q=0 and calculate the gradient $m=\frac{\partial Q\left(\sigma_{m},\rho ,r,k\right)}{\partial \sigma }$.
  3. Compute $\Delta \lambda =\frac{F_{B}}{n_{B}^{T}Hm_{B}+H_{pB}}$ and the stresses at point *C*: $\sigma_{C}=\sigma_{B}-\Delta \lambda Hm_{B}$, where $\sigma_{B}$ is the elastic test point; H is elasticity tensor; and $H_{g}$ is effective plastic modulus (*g* is generic variable, g=p for hardening and g=c for softening).
  4. Update the equivalent plastic strain $\epsilon_{p}\left(\epsilon_{f}\right)$ in hardening (softening) and the strength parameter k(c) during the hardening(softening).

- Beginning the implicit backward-Euler method:
  5. Calculate *F* and n at the current point *C*.
  6. Minimize the potential for Q=0 and calculate the gradient m.
  7. Calculate the residual $r_{0}=\sigma_{C}-\left\{\sigma_{B}-\Delta \lambda Hm_{C}\right\}$.
  8. Compute the change of the plastic multiplier:
    $$\delta \lambda =\frac{F_{C_{0}}-n_{C}^{T}{\left[I+\Delta \lambda \;H\;\frac{\partial m}{\partial \sigma }{|}_{C}\right]}^{-1}r_{0}}{n_{C}^{T}{\left[I+\Delta \lambda \;H\;\frac{\partial m}{\partial \sigma }{|}_{C}\right]}^{-1}\;H\;m_{C}+H_{pC}}$$
    and then change the stresses
    $$\delta \sigma =-{\left[I+\Delta \lambda \;H\;\frac{\partial m}{\partial \sigma }{|}_{C}\right]}^{-1}\left\{r_{0}+\delta \lambda \;H\;m_{C}\right\}$$
  9. Update the stresses at the point *C*: $\sigma_{C_{n}}=\sigma_{C_{0}}+\delta \sigma$, then calculate the changes in plastic multiplier at point *B* (Figure 7): $\Delta \lambda_{n}=\Delta \lambda_{0}+\delta \lambda$
  10. Update the equivalent plastic strain $\epsilon_{p}\left(\epsilon_{f}\right)$ and the strength parameter k(c) during the hardening(softening).
  11. Repeat the procedure from step 5 until r and *F* are below a certain tolerance.

## 6. Calibration

The parameter $\alpha_{f}$ used to express the function $\rho (\sigma_{m})$ is the first parameter that can be used to identify the failure envelope *F*. This parameter is set to 2.5. The second parameter $\beta_{f}$ [in the hardening term] used to define the dependence of the resistance is a power function of the parameter *k*. This setting offers the possibility of concrete to hardening in tension. As a first approximation, the parameter $\beta_{f}$ is constant and equal to the parameter $\alpha_{f}$. These parameters $a_{f}$ and $b_{f}$ are identified manually by trials and errors until an accurate fitting of the experimental data is achieved. The selected combination should offer the lowest absolute error (Root Mean Square (*RMS*)) between the measured shear stresses and those defined by the criterion at the same pressures. This analysis is repeated for the plastic potential. Due to non-associatedness law, the potential has a slightly different form of the failure envelope. Three parameters ($a_{q}$, $b_{q}$, and $\alpha_{q}$) are then identified. For the plastic potential, its normal direction is more important than its magnitude. The potential plastic used for the concrete must have the following characteristics [7]:

1. It should promote a positive change in volumetric plastic in the region of positive pressure related to the mode of crack opening.
2. It should promote a change in plastic form in the region of negative pressure related to the mode of cracking or splitting in shear compression.

**Figure 8:**
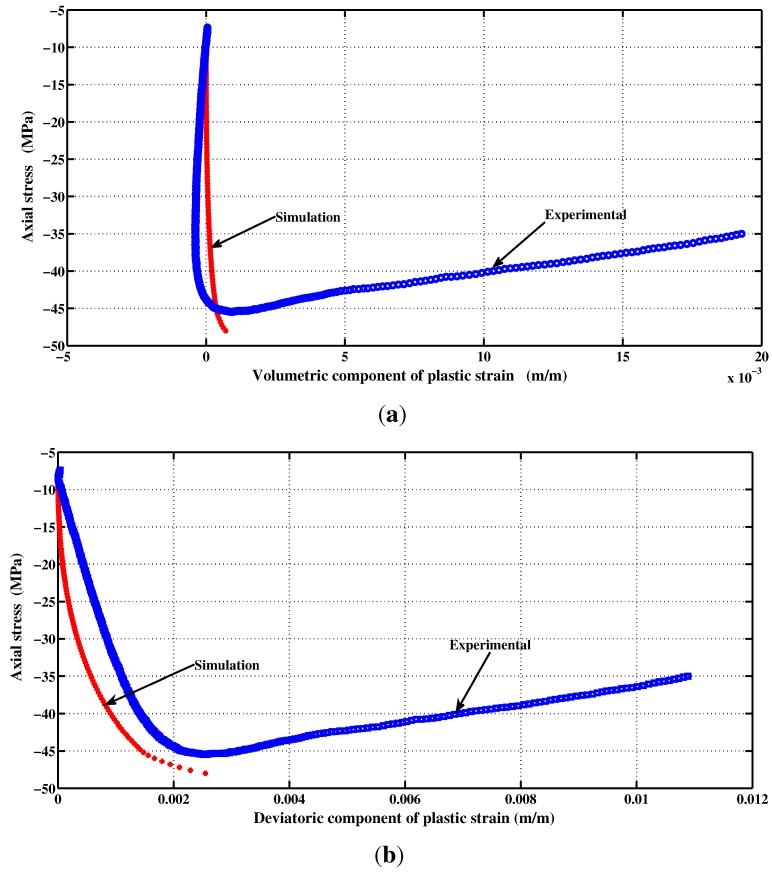
Volumetric and deviatoric plastic components correlations for data obtained by simulation and experiment. (**a**) Volumetric; (**b**) Deviatoric.

**Figure 9:**
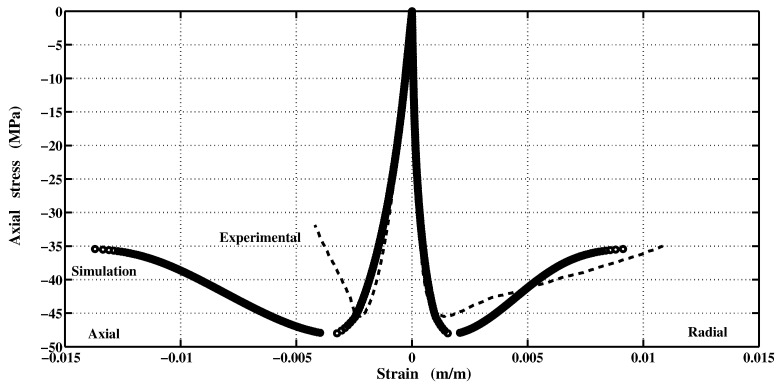
Numerical and experimental stress-strain curve in axial compression.

The elastic-plastic model developed by Kang [7] makes it possible to reach the same specifications on the behavior of concrete. The developed plastic potential expression can now answer the two features mentioned above through the parameter $\alpha_{q}$. By setting values of $\alpha_{q}$, it is possible to get a pronounced curvature of the function $\rho (\sigma_{m})$. To identify the last two parameters $a_{q}$ and $b_{q}$, the inverse approach must be used. It consists in setting manually the values of parameters $a_{q}$ and $b_{q}$, and then simulate a failure mode to observe the permanent components of volumetric and deviatoric deformations. The model is programmed in the Matlab software and the loading is controlled by stress and by imposing increments in axial direction only. From permanent deformations, the plastic volumetric and deviatoric strains components are calculated and then compared with those measured in the laboratory. Figure 8b shows the results for a simple compression test. According to the simulated data, the ratio of the deviatoric and the volumetric components is preserved almost up to the failure. However, during the hardening process, the plastic volumetric strain component has greater amplitude. Figure 9 shows numerical and experimental correlation obtained through a simple compression test. If the correlation is deemed to be satisfied, the values of $\alpha_{q}$, $a_{q}$ and $b_{q}$ parameters are considered acceptable. The values of the optimized parameters of the proposed triaxial concrete model are: $\alpha_{f}=2.5$; $a_{f}=3.8602$; $b_{f}=6.0$; and $\beta_{f}=2.5$ (Figure 2). The set of parameters for the plastic potential are: $\alpha_{q}=6.50$; $a_{q}=8.10$; $b_{q}=12.20$; and $\beta_{q}=6.50$.

## 7. Numerical Experiments for Various Loading Scenarios

**Figure 10:**
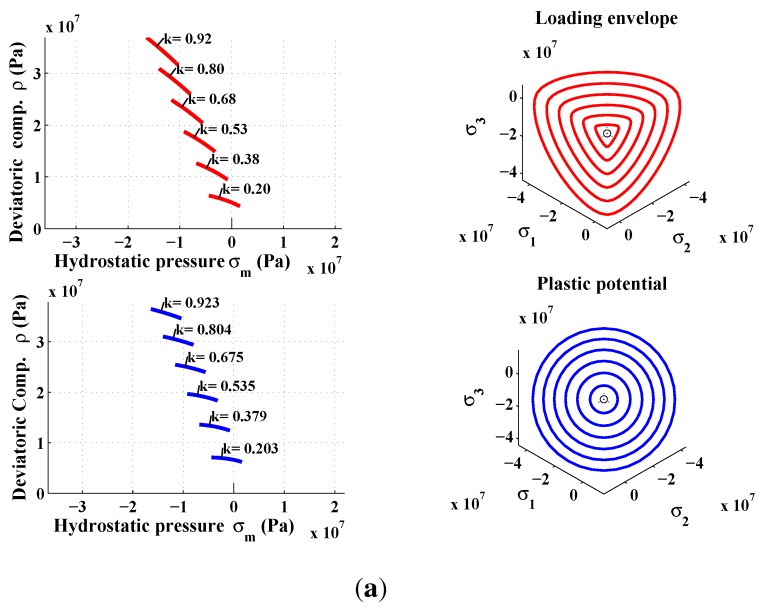

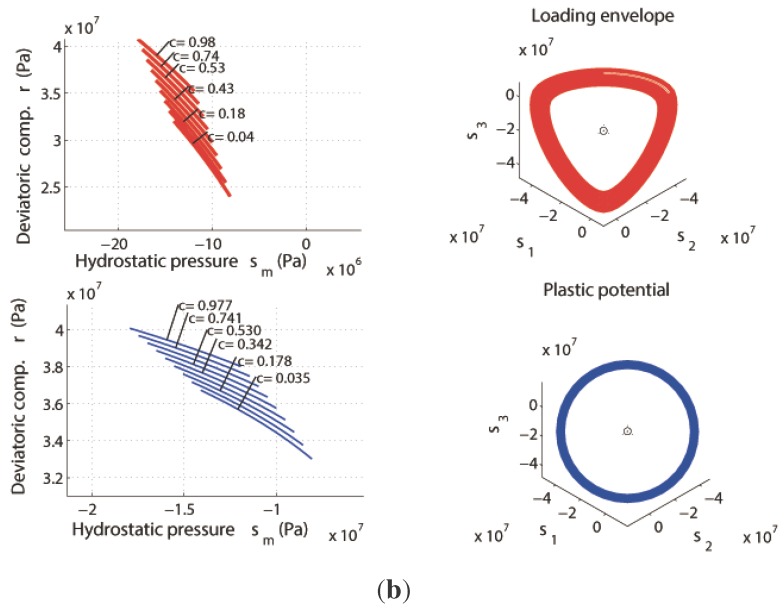
Hardening and softening stages in uniaxial compression test. (**a**) Hardening; (**b**) Softening.

**Figure 11:**
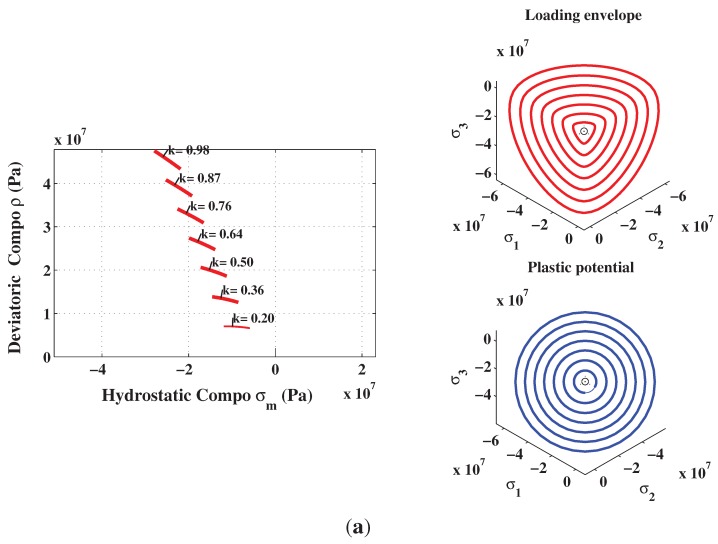

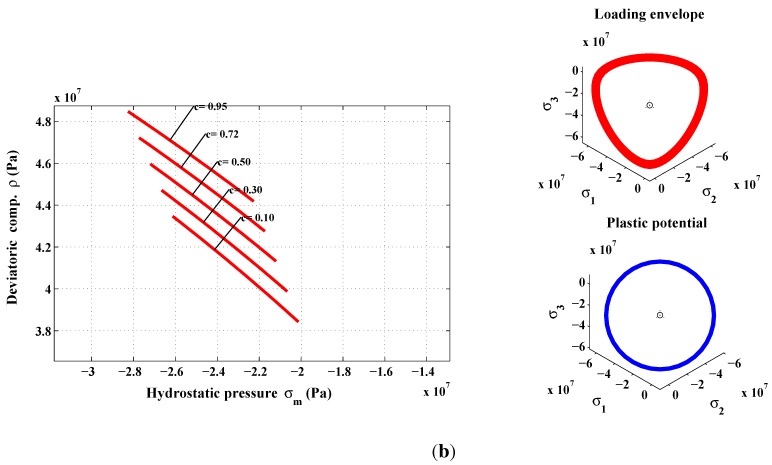
Hardening and softening stages in numerical triaxial compression test. (**a**) Hardening; (**b**) Softening.

**Figure 12:**
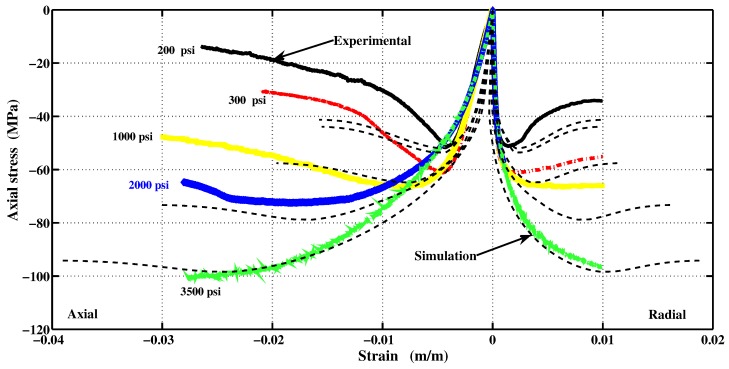
Comparison between numerical and experimental results for concrete under triaxial compression at different confinement pressures.

The proposed constitutive model was implemented in Matlab. The capability and performance of the present model is validated by comparing the predicted values with experimental data [36]. Uniaxial and triaxial compressions, as well as direct shear scenario are also applied. The comparisons between numerical and experimental results for concrete under uniaxial compression, in both axial and radial directions, are presented in Figure 9 and Figure 11. The correlation is very reasonable. The hardening regime is similar to that measured experimentally. At the end of the softening, the model slightly overestimates the axial stress. The surfaces of residual loading of Figure 5 are not close enough for this type of loading. The parameters $a_{f}$ and $b_{f}$ depending on the cohesion parameter *c* maybe varied to improve the fitting. As can be observed in Figure 10(a) and Figure 10(b), the failure criterion and the plastic potential go through the same strength parameter *k* (or cohesion parameter *c*) as explained in Section 5.1. Furthermore, comparison between numerical and experimental results for concrete under triaxial compression and various confinement levels are presented in Figure 11 and Figure 12. The correlation is acceptable for both axial and radial directions. The numerical results are very close to the experimental ones.

**Figure 13:**
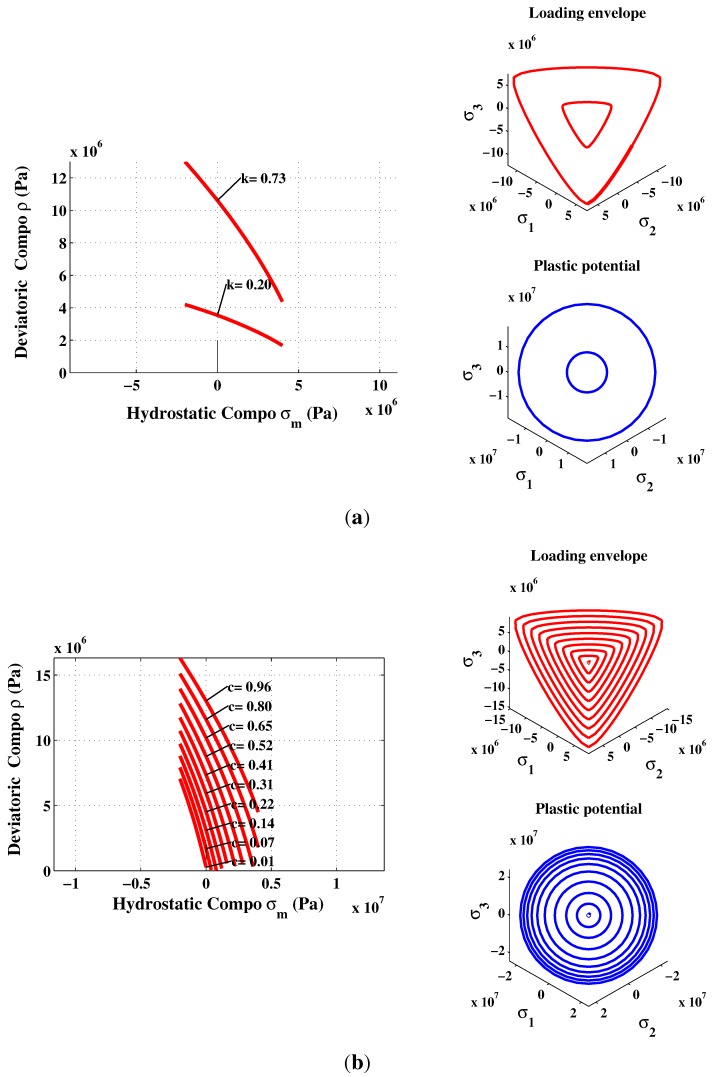
Hardening and softening stages in numerical pure shear test. (**a**) Hardening; (**b**) Softening.

**Figure 14:**
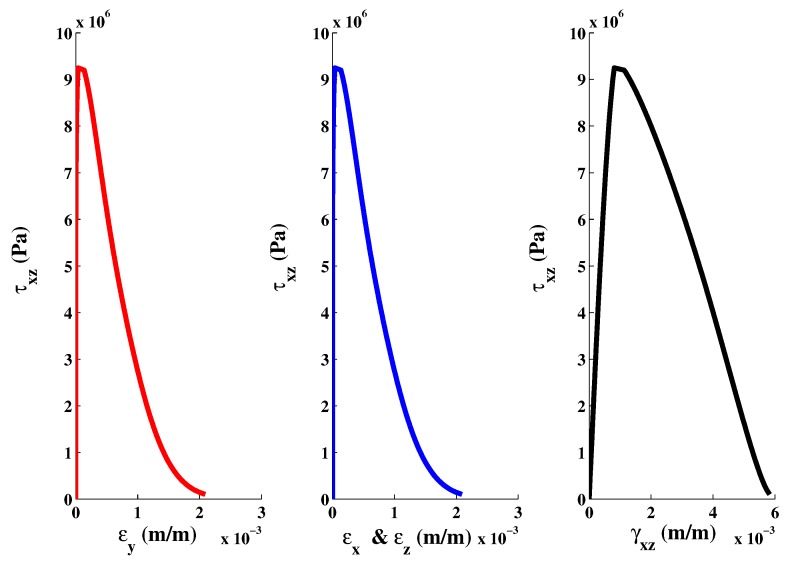
Stress-strains curves in numerical pure shear test.

The simulation of a pure shear is presented in Figure 13 and Figure 14. There is however no experimental curve for concrete that can be used to validate this trend.

**Figure 15:**
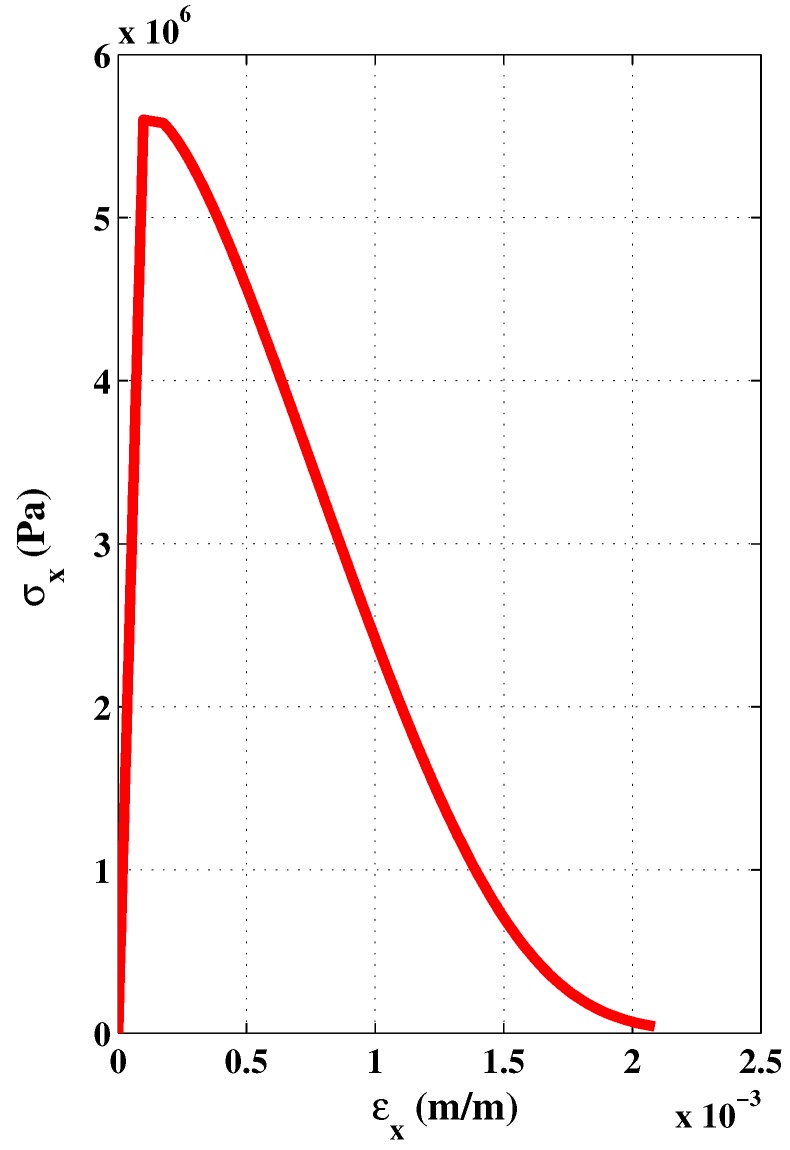
Stress-strain curve from numerical triaxial tension test.

**Figure 16:**
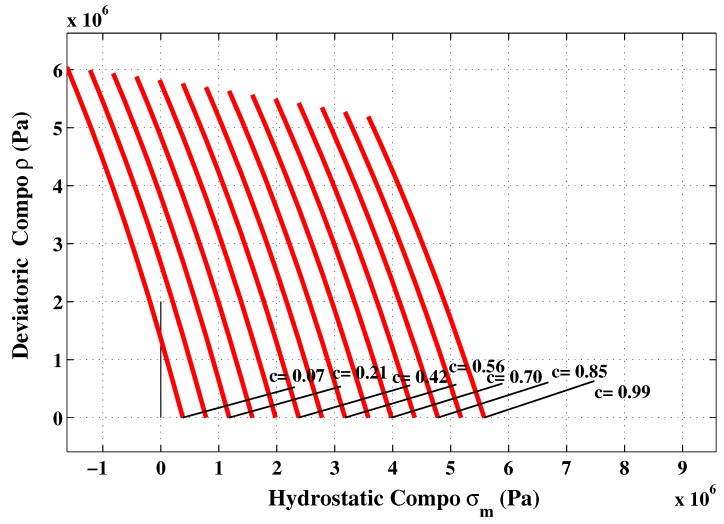
Softening stage in numerical triaxial tension test.

Figure 15 and Figure 16 show the simulated stress-strain curves for triaxial tension. The observed failure criterion is close to that of Rankine. Based on that Rankine criterion, the model accurately estimates the strength of the material. The model assumes that the material does not present hardening stage. The main crack propagation is failing its residual strength as if the material behaved fragile.

## 8. Conclusions

A triaxial elastoplastic constitutive law for concrete under inelasticity framework was developed and validated. The model captures the entire response spectrum in tension as well as in shear within a unified formulation. The model prediction showed a reasonable agreement with experimental response and failure data. Thus, the proposed constitutive theory has considerable potential for finite element analysis of unreinforced and reinforced concrete structures. The generic calibration map was mainly attributed to the intrinsic scatter of the experimental results. Notwithstanding this scatter, the model offers flexibility against specific experimental datasets, which allowed easily recalibrating the model and adapting it to technical requirements.

The model was composed by:

1. A five parameters loading surface, which was adapted and calibrated by a simple procedure.
2. Uncoupled hardening and softening functions following the accumulation of plastic strain and ductility evolution.
3. A new ductility function was proposed and fitted experimentally.
4. A new nonlinear plastic potential function was developed and calibrated using database of test results (uniaxial compression).
5. As the failure criterion and plastic potential do not undergo the same stress states, a projection procedure has been adopted and applied to the concrete case. The calculation of normal is accurate and verified through numerical simulations.
